# Measuring TechnoWellness and Its Relationship to Subjective Well-Being: the Mediating Role of Optimism

**DOI:** 10.11621/pir.2021.0311

**Published:** 2021-09-30

**Authors:** Octav S. Candel

**Affiliations:** Alexandru Ioan Cuza University of Iasi, Iasi, Romania

**Keywords:** Technology, positive psychology, satisfaction with life, positive affect, negative affect

## Abstract

**Background:**

The relationship between technology use and subjective well-being is controversial, with recent research suggesting both positive and negative links. Thus, it is important to determine whether using technology actually leads to flourishing and well-being. To do so, the construct of TechnoWellness, which includes both adaptive and maladaptive modes of using technology, could be particularly useful. Moreover, the mechanisms that account for these relationships have been insufficiently explored.

**Objective:**

With this study, we had two objectives. First, we aimed to test the relationships between TechnoWellness and the three components of subjective well-being (positive affect, negative affect, and satisfaction with life). Secondly, we aimed to investigate whether optimism mediates those relationships.

**Design:**

A total of 366 participants (122 men and 244 women, mean age = 22.34 years old) took part in this study. In this cross-sectional investigation, we measured TechnoWellness, optimism, positive and negative affect, satisfaction with life, and some demographic variables, using an online form. To verify our aims we used structural equation modeling (SEM) with mediation.

**Results:**

Among the positive factors of TechnoWellness, only using technology for physical activity was directly associated with satisfaction with life. Among the negative factors, the stress caused by technology use was directly associated with negative affect. Optimism mediated the relationships between these two factors (using technology for physical activity and stress caused by technology use) and all the components of subjective well-being.

**Conclusion:**

Using technology to foster wellness is related to actual subjective well-being. However, the purpose, frequency, and type of its use are important elements to be considered when studying its effects.

## Introduction

Is technology use related to subjective well-being? A growing body of research, having its roots in the frameworks of positive psychology and positive technology, indicates an affirmative answer to this question. Using technology can lead to improving various levels of human functioning, such as fostering positive emotions, happiness, strength, resilience, and stronger interpersonal relationships (Botella et al., 2012). However, to fully comprehend this relationship, one must find the different mechanisms that can affect it. Moreover, not all forms of technology are useful and beneficial to individuals. The development of technology has also led to the advent of new issues that can negatively impact one’s level of well-being (Neverkovich et al., 2018; Tarafdar, Cooper, & Stich, 2017).

With this study, we had two aims. First, we set out to test the relationships between TechnoWellness, “a mode of interacting with technology that maximizes its potential to enhance health and well-being” (Kennedy, 2014, p. 114) and subjective well-being. Second, we sought to investigate whether optimism mediates the relationship.

Technology use is a broad term that can include every kind of interaction between humans and machines. Thus, it can refer to the use of mechanical tools in factory settings as well as the use of the latest information and communication technologies (ICTs). No matter what the setting, the proponents of the positive technology framework consider that technology can “improve the quality of the personal experience, which in turn serves to promote wellness and generate resources and strengths in individuals” (Botella et al., 2012, p. 78). This same framework led to the proposal of a new construct, that of TechnoWellness (Kennedy, 2014; Kennedy & Baker, 2016). Rooted in the Indivisible Self Model of Wellness (IS-Wel; Myers & Sweeney, 2005), this construct can offer insight into how some domains of technology use affect subjective well-being.

The need to measure TechnoWellness led to the development of the TechnoWell-ness Inventory (TWI; Kennedy & Baker, 2016). An exploratory factor analysis revealed a five-factor structure, with three factors representing adaptive ways to use technology (for leisure, physical activity, or vocational purposes), and two factors representing maladaptive effects of technology use (stress and excessive use). Some important advantages that TechnoWellness has over other constructs were derived from the framework of positive technology. These include incorporating more facets of the person’s character (it includes questions related to the social self, creativity, coping, spirituality, and physical health), while also taking into account the negative outcomes of technology use (Kitson, Prpa, & Riecke, 2018).

However, this construct has some limitations, given that it has only been empirically tested in a few studies. To our knowledge, TechnoWellness has been the subject of only two studies, where it presented significant associations with holistic wellness and happiness (Kennedy & Baker, 2016; Shawaqfeh & Almahaireh, 2019). Nonetheless, the concept of TechnoWellness shares major similarities to the more general idea of technology use for positive outcomes (Kitson et al., 2018). Moreover, the relationship between different forms of technology use and subjective well-being has been more thoroughly studied.

Subjective well-being (SWB) represents “a person’s cognitive and affective evaluations of his or her life” (Diener, Lucas, & Oishi, 2002, p. 63). It has three main components: positive affect, negative affect, and satisfaction with life (Andrews & Withey, 1976). Thus, for individuals to consider that they have high levels of SWB, they must show high levels of positive affect and satisfaction with life, and low levels of negative affect.

Previous studies have shown that, when used in moderation, digital technologies can lead to higher levels of mental well-being for adolescents (Przybylski & Weinstein, 2017). Moreover, Facebook use and the number of Facebook friends have been associated with psychological well-being for those between 18 and 34 years old (Chan, 2018). Although there is a persistent concern about the maladaptive effects of some technology use, recent research suggests that the negative effects are small or inconsistent (Orben & Przybylski, 2019a; Orben & Przybylski, 2019b). Also, for older individuals, technology use and positive attitudes towards technology are related to higher levels of well-being (Sims, Reed, & Carr, 2017; Zambianchi & Carelli, 2018).

In the work domain, the use of technology has been associated with higher well-being through enhanced work/life balance, greater autonomy, and more effective communication (ter Hoeven & van Zoonen, 2015). Finally, various positive psychology interventions targeting subjective well-being have been implemented through the use of positive technologies (Botella, Baños, & Guillen, 2017; Diefenbach, 2018).

However, one should also take into account the negative impact of technology use on subjective well-being. Problematic use of smartphones was found to be related to lower global SWB and higher negative emotions (Chan, 2018; Hornwood & Anglim, 2019). A meta-analysis of 28 studies also showed that problematic internet use is negatively related to SWB (Çikrıkci, 2016). Also, social media addiction negatively impacted life satisfaction in a sample of students (Hawi & Samaha, 2016). Finally, techno-stress (“stress that individuals experience due to their use of Information Systems,” Tarafdar et al., 2017, p. 7) negatively impacts well-being in a variety of domains (Dragano & Lunau, 2020; Nimrod, 2018).

Thus, the way technology influences subjective well-being becomes highly dependent on the frequency of use, on the way the people use it, and on what type of technology they use (Cotten, 2018). Using a concept such as TechnoWellness, which incorporates both the positive and the negative aspects of technology use, allows us to test this complex relationship between technology and SWB. More precisely, we decided to explore how the use of technology to maximize wellness and well-being is actually related to well-being. We set forth the following hypothesis:

H1: TechnoWellness is associated with subjective well-being:

H1a: Using technology for leisure, physical activity, and vocational purposes is positively associated with positive affect and satisfaction with life, and negatively associated with negative affect.

H1b: Excessive use of technology and stress are negatively associated with positive affect and satisfaction with life, and positively associated with negative affect.

Previous empirical studies found that personality factors are the most important predictors of SWB (Daukantait & Zukauskiene, 2012). Some of them impact one’s perspective on current or future events, thus affecting life satisfaction and its sustainability (Duy & Yildiz, 2019). One stable personality trait that greatly contributes to an individual’s perception of future events is optimism, which is defined as a generalized tendency to expect positive outcomes in life (Scheier & Carver, 1985). Because optimists believe that their actions can lead to various positive outcomes, they use elevated problem-focused coping and better planning, and display a higher general expectation that they will reach their goals (Bailey, Eng, Frisch, & Snyder, 2007).

There is a high level of consensus regarding the link between optimism and the components of SWB, with the majority of studies showing a significant and positive relationship. Previous research showed that optimism is related to global SWB in various cultures (Duy & Yildiz, 2019; Satici, 2019; Utsey, Hook, Fisher, & Belvet, 2008; Yu & Luo, 2018), as well as to its components, such as life satisfaction (Bailey et al., 2007; Daukantait & Zukauskiene, 2012; Razaei & Khosroshahi, 2018) or positive and negative affect (Esteve et al., 2018; Hajek & Koning, 2019).

The use of technology has brought humanity unprecedented levels of progress. Thus, people maintain an implicit association between technology and success that leads to higher levels of optimism in the fields where technology is used (Clark, Robert, & Hampton, 2016). Although this “technology effect” can have its maladaptive outcomes (such as biased decision-making, as pointed out by Clark et al., 2016), we consider that it can also lead to positive outcomes. Past studies have shown that Techno-Wellness is positively related to optimism (Shawaqfeh & Almahaireh, 2019), and that some interventions delivered through positive technology can lead to an improvement in future expectations (Enrique, Breton-Lopez, Molinari, Banos, & Botella, 2018). On the contrary, maladaptive use of ICTs was related to lower optimism (Guo, You, Gu, Wu, & Xu, 2020). Thus, there is evidence that technology use is related to optimism, and that optimism is related to SWB. Based on this, we proposed our second major hypothesis:

H2. Optimism may mediate the relationship between TechnoWellness and subjective well-being.

## Methods

### Participants

Three hundred sixty-six participants (122 men and 244 women) took part in this study. The mean age in our sample was 22.34 years old (SD = 3.36, Min. = 18 years old, Max. = 29 years old). Three hundred forty-nine participants reported they were heterosexual, four participants reported they were homosexual, and 13 participants reported they were bisexual. Among the participants, 283 had finished high school at the time of the study, 64 had a bachelor’s degree, and 19 had a master’s degree.

### Procedure

This study was approved by the University’s ethics board. The participants were recruited from students enrolled in various bachelor’s degree programs. Participation was voluntary and was rewarded with course credit. All the data was gathered using an online form containing the questionnaires for TechnoWellness, optimism, subjective well-being, and demographic information.

### Measures

#### TechnoWellness

TechnoWellness was measured using the TechnoWellness Inventory (Kennedy & Baker, 2016). Its items describe specific interactions with technology that could have a significant impact on a person’s wellness in either positive or negative ways. The scale consists of 76 items rated on a scale from 1 (strongly disagree) to 4 (strongly agree). Some items are reverse coded.

The instrument measures five factors of TechnoWellness: 1) leisure (39 items, *e.g.,* “I use technology to connect with new friends I have met in person”; Cronbach’s alpha = .83); 2) stress (18 items, *e.g.,* “I worry about my ability to use new technologies”; Cronbach’s alpha = .88); 3) vocational use (7 items, *e.g.*, “Using computers and other technology helps me to feel more in control of my work”; Cronbach’s alpha = .67); 4) physical activity use (7 items, *e.g.*, “I go online to find information about healthy nutrition”; Cronbach’s alpha = .85); and 5) excessive use (4 items, *e.g.*, “I sometimes do not get enough sleep because of the time I spend online”; Cronbach’s alpha = .76).

#### Optimism

Optimism was measured with the Life Orientation Test-Revised (LOT-R; Scheier, Carver, & Bridges, 1994). The scale contains 10 items (three for optimism, three for pessimism, and four that are not computed in the final score), measured on a scale from 1 (total disagreement) to 7 (total agreement). The authors recommended the inclusion of the four filler items to disguise the purpose of the questionnaire. The items for pessimism are reverse coded. For this study, the scale reported a good internal consistency (Cronbach’s alpha = .80).

#### Emotional Response

To measure the positive and negative emotional components of subjective well-being, we utilized the 10 item short-form of the Positive and Negative Affectivity Scale developed and validated by Thompson (2007). Participants rated five adjectives for positive affect (Active, Determined, Attentive, Inspired, and Alert) and five for negative affect (Upset, Hostile, Ashamed, Nervous, and Afraid), when asked how they felt recently, using a scale from 1 (never) to 5 (always). For the Positive Affect factor, Cronbach’s alpha was .69. For the Negative Affect factor, Cronbach’s alpha was .66.&

#### Satisfaction with Life

Satisfaction with life was measured with the Satisfaction with Life Scale (SWLS, Diener, Emmons, Larsen, & Griffin, 1985). This is a 5-item scale designed to measure global cognitive judgments of one’s subjective well-being. The participants indicated how much they agreed or disagreed with each of the 5 items, using a 7-point scale that ranged from 1 (strongly disagree) to 7 (strongly agree). Higher scores indicated higher satisfaction with life. The Cronbach’s alpha for this study was .82.

Additional questions dealt with the participants’ demographic characteristics (age, sex, sexual orientation, and level of studies) and their use of technology (how comfortable they are with the use of technology and how much they use it). The latter was measured on a two item scale, from 1 (not that much) to 5 (a lot).

## Results

First, we aimed to assess the level of technology use in our sample. We found that the mean response to the item “How much do you use technology in your life” was 4.65 (SD = .58), indicating high usage. Also, the participants seemed to be comfortable with using technology, with a mean score of 4.56 (SD = .67).

Second, we computed the descriptive analyses and Pearson product-moment correlation between the study’s variables. The results of these analyses can be seen in *Table 1.* We found that, among the dimensions of TechnoWellness, only the stress derived from technology use correlated significantly and negatively with optimism. In regard to the associations with SWB, using technology for leisure activities correlated significantly and positively with negative affect. The stress derived from technology use correlated significantly and positively with negative affect. Finally, the use of technology for physical activity correlated significantly and positively with satisfaction with life. The level of optimism correlated significantly with all three dimensions of SWB, being positively associated with positive affect and satisfaction with life, and negatively associated with negative affect.

**Table 1:** Means, standard deviations, and correlations among the study’s variables

	M	SD	1	2	3	4	5	6	7	8
1. Leisure use	2.56	.35	1							
2. Vocational use	3.04	.54	.36^**^	1						
3. Physical activity use	2.18	.77	.46^***^	.13^*^	1					
4. Stress	1.98	.57	.32^***^	–.09	.27^***^	1				
5. Excess use	2.23	.64	.26^***^	.04	.10	.24^***^	1			
6. Optimism	2.40	.80	–.04	–.02	.04	–.19^***^	–.07	1		
7. Positive affect	3.46	.68	.07	.08	.05	–.03	–.06	.36^***^	1	
8. Negative affect	2.62	.82	.12^*^	–.09	.08	.36^***^	.03	–.37^***^	–.07	1
9. Satisfaction with life	4.75	1.15	.10	.07	.16^**^	–.05	.07	.51^***^	.29^***^	–.28^***^

Note: *** p < .001, **p < .01, * p < .05

Although we did not find significant correlations among all the proposed predictors, the mediator, and all the proposed outcomes, we still proceeded to test the proposed mediation model. The analyses were in line with recent guidelines that support this approach (Rucker, Preacher, Tormala, & Petty, 2011). We used structural equation modeling (SEM) and created a model where the dimensions of TechnoWellness were introduced as predictors, the dimensions of SWB as outcomes, and optimism as a mediator. One of the main advantages of this kind of analysis is that it allows the use of multiple predictors, multiple outcomes, and the simultaneous investigation of the relations between them. In this model, we additionally controlled for age, sex, and comfort with the use of technology, and we allowed the control variables to correlate with the predictors.

**Figure 1.**
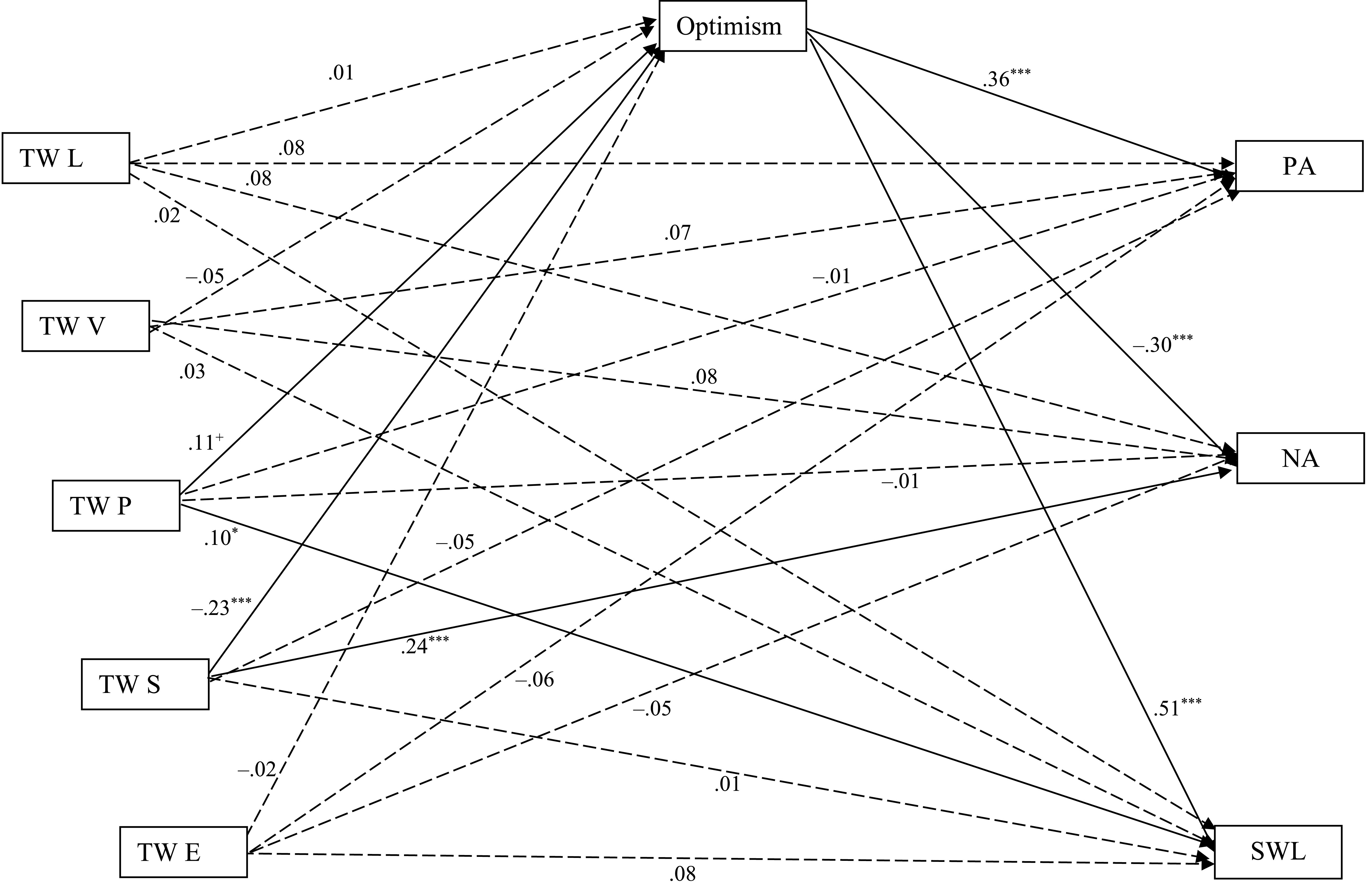
Standardized direct effect from TechnoWellness to SWB and optimism and from optimism to SWB

Note. Significant paths are indicated with continuous lines. TW L = TechnoWellness leisure use; TW V = TechnoWellness vocational use; TWP = TechnoWellness physical activity use; TW S = TechnoWellness stress; TW E = TechnoWellness excessive use; PA = Positive Affect; NA = Negative Affect; SWL = Satisfaction with life; p = .06; ^*^ p < .05; ^***^ p < .001.

This model presented good fit indices (χ2 = 20.82, df. = 8, p = .008, CFI= .98, GFI= .99, RMSEA = .06, SRMR = .03). By analyzing the relationships among the variables (see *Figure 1*), we found that using technology for physical activity has a significant direct effect on satisfaction with life. Also, the stress caused by the use of technology had a significant positive effect on negative affect. Moreover, higher use of technology for physical activity was related, although just barely, to higher levels of optimism, and the stress caused by the use of technology was significantly and negatively related to optimism. Optimism was significantly related to all the components of SWB.

Analyzing the indirect effects (*Table 2*) provided us with further significant relationships. First, using technology for physical activity was, through optimism, indirectly associated with all the components of SWB (positive affect: β = .04, 95% CI [.007; .08]; negative affect: β = –.03, 95% CI [–.07; –.006]; satisfaction with life: β = .05, 95% CI [.008; .11]). Second, the stress caused by the use of technology was, through optimism, indirectly associated with the components of SWB (positive affect: β = –.08, 95% CI [–.13; –.05]; negative affect: β = .07, 95% CI [.04; .010]; satisfaction with life: β = –.11, 95% CI [–.17; .07]).

**Table 2:** Standardized indirect effects from TechnoWellnes to SWB through optimism

	Beta	S.E.	p	95% CI
Leisure use → Optimism → Positive Affect	.01	.02	.91	[–.03; .04]
Leisure use → Optimism → Negative Affect	–.01	.02	.91	[–.03; .03]
Leisure use → Optimism → Satisfaction with life	.01	.03	.92	[–.05; .06]
Vocational use → Optimism → Positive Affect	–.02	.02	.24	[–.05; .01]
Vocational use → Optimism → Negative Affect	.01	.01	.28	[–.01; .04]
Vocational use → Optimism → Satisfaction with life	–.03	.02	.27	[–.07; .01]
Physical activity use → Optimism → Positive Affect	.04	.02	.04	[.01; 08]
Physical activity use → Optimism → Negative Affect	–.03	.01	.04	[–.06; –.01]
Physical activity use → Optimism → Satisfaction with life	.05	.03	.05	[.01; .11]
Stress → Optimism → Positive Affect	–.08	.02	.001	[–.12; –.05]
Stress → Optimism → Negative Affect	.07	.01	.001	[.04; .10]
Stress → Optimism → Satisfaction with life	–.11	.03	.001	[–.17; –.07]
Excess use → Optimism → Positive Affect	–.01	.02	.67	[–.04; 02]
Excess use → Optimism → Negative Affect	.01	.01	.67	[–.02.03]
Excess use → Optimism → Satisfaction with life	–.01	.03	.68	[–.06; .03]

## Discussion

Subjective well-being is related to various factors, such as personality, social relationships, or contextual and environmental agents (Kobylinska, Zajenkowski, Lewczuk, Jankowski, & Marchlewska, 2020; Nartova-Bochaver, Mukhortova, & Irkhin, 2020; Siedlecki, Salthouse, Oishi, & Jeswani, 2014; Zotova & Karapetyan, 2018). In recent years, the changes specific to modern life have brought into discussion a new potential pathway to human flourishing and well-being. The theoretical frameworks of positive psychology and positive technology speak about the advantages of using technology to improve quality of life and well-being (Botella et al., 2012).

However, the use of technology can also lead to maladaptive outcomes (Çikrıkci, 2016; Neverkovich et al., 2018; Tarafdar et al., 2017). Moreover, even in the case of positive relationships between technology use and well-being, their mechanisms have been insufficiently explored. With this study, we aimed to account for these limitations by testing the links between TechnoWellness, or the use of technology to maximize wellness and well-being, and actual subjective well-being, and by proposing a potential mediator of the relationship in the form of optimism.

First, we found that among the positive factors of TechnoWellness, only using technology for physical activity had a direct link with one facet of SWB, namely satisfaction with life. Specifically, people who used technology for physical activity more often (by using different mobile applications to track their activity, their calorie intake, or by searching for the best possible fitness exercises on the Internet) also reported higher levels of satisfaction with life. In terms of indirect effects, the same factor of TechnoWellness had positive associations with positive affect and satisfaction with life, and negative association with negative affect. The people who used technology to augment their physical training reported higher SWB scores, their level of optimism mediating this relationship. These results confirmed previous findings which reported that regular physical activity and exercise enhance subjective well-being (Jeoung, 2020; Kim, Kubzansky, Soo, & Boehm, 2017; Lawton, Brymer, Clough, & Denovan, 2017; Mandolesi et al., 2018; Maugeri et al., 2020).

Moreover, with our study, we suggest that technology plays an important part in this relationship. Technology offers new opportunities for maintaining a healthy lifestyle through regular exercise and more opportunities for control (Milani & Franklin, 2017) and thus it relates positively to more satisfaction with life. Also, using technology for physical activity was associated with higher levels of optimism. By taking into account the “technology effect” (Clark et al., 2016), people might consider that technology improves their chances of success in keeping a healthy lifestyle, which affects their tendency to expect positive outcomes in the future. Furthermore, our study confirmed the previous findings that optimism was associated with SWB (Duy & Yildiz, 2019; Satici, 2019; Utsey et al., 2008; Yu & Luo, 2018). Optimists are more confident in their abilities, perseverant in their efforts, and consistent in their interests, characteristics that are important for flourishing and subjective well-being (Datu, McInerney, Zemojtel-Piotrowska, Hitokoto, & Datu, 2020; Oriol, Miranda, Bazan, & Benavente, 2020). Hence, it can be stated that this result is consistent with that of previous studies, which suggested that optimism may play a role in the relationship between using technology for physical activity and SWB.

Second, we found the opposite relationship between the stress caused by the use of technology and SWB. Stress seemed to affect the components of SWB by reducing optimism. Our findings support the results of studies suggesting that stress in general, including techno-stress, gravely impacts SWB (Choi & Lim, 2016; He, Turnbull, Kirshbaum, Phillips, & Klainin-Yobas, 2018; Nimrod, 2017; Smith & Yang, 2017). Using technology can lead to overload, uncertainty, insecurity, and feelings of invasion, and it entails a constant need to adapt to new developments and discoveries (Nimrod, 2017; Tarafdar et al., 2017). According to our study, all these negative responses to technology use are associated with lower levels of optimism (people might stop expecting positive outcomes because they feel overwhelmed by technology) and, through it, with lower satisfaction with life, lower positive affect, and higher negative affect.

The other two positive factors of TechnoWellness – using technology for leisure and vocational purposes – did not present significant associations with SWB. On the one hand, this shows that it is important to take into account the purpose for using technology when discussing its positive or negative relationships with SWB. On the other hand, using technology for leisure and vocational purposes might still have an indirect association with SWB. For this reason, future studies need to take into account other potential mediators, such as self-efficacy and social support or authenticity, factors that have previously been connected to flourishing and well-being (Liu et al., 2018; Reinecke & Trepte, 2014; Rivera et al., 2018; Siedlecki et al., 2014).

We also found no relationship between excessive technology use and SWB, a result which contradicts previous studies (Chan, 2018; Çikrıkci, 2016). However, it is important to take into account that in this study, we were only measuring the perceived use of technology, not the actual use of it. Previous studies showed only weak correlations between these two measures, suggesting that they may widely differ (Sewall, Bear, Merranko, & Rosen, 2020). This difference might represent a potential limitation of the TechnoWellness model and should be further studied.

## Conclusion

This study had multiple strengths. Firstly, from a theoretical standpoint, it showed that the construct of TechnoWellness (Kennedy, 2014; Kennedy & Baker, 2016) was related to subjective well-being. Although we did not find significant relationships among all of its components and SWB, our results strongly suggested that using technology to enhance health and well-being is actually related to higher levels of wellbeing. Thus, our study adhered to the framework of positive technology by showing that using technology by itself can help the development of wellness.

Also, TechnoWellness takes into account the potentially damaging effects of technology use. This study found a significant and negative relationship between stress caused by technology use and SWB. Taken together, these results offer support for the idea that the purpose, frequency, and type of technology use are important elements to be considered when studying its effects. Moreover, we proposed a mechanism to explain these relationships. We found that the associations between TechnoWellness and SWB were mediated by optimism. Thus, our results support the inclusion of other potential mediators in future research.

## Limitations

This study was not without its limitations. First, the paper used cross-sectional data in the analyses. Thus, the results are more associative than inferential. Future longitudinal or experimental studies are needed to draw more exact conclusions regarding the predictive power of TechnoWellness on subjective well-being.

Second, we used a convenience sample composed of university students. While the youth population is most closely associated with technology use, it would be important to explore the same variables in an older sample. Additionally, nowadays people over 40 years old are using technology more than they did a few years back, but some effects of technology use might be unique among their age group (such as techno-stress).

Third, we used only self-report scales. We already mentioned that perceived technology use might be different from actual technology use, so future studies should take into account the use of more objective measures.

Finally, a study by Sewall and colleagues (2020) suggested that the respondents’ well-being was associated with the degree of inaccuracy in their estimated digital technology use. This finding shows that a bi-directional relationship between technology use and SWB should be taken into account. Future studies could be interested in testing whether different levels of well-being also impact the way people report technology use, especially in the case of perceived use.
